# Object Recognition and Localization: The Role of Tactile Sensors

**DOI:** 10.3390/s140203227

**Published:** 2014-02-18

**Authors:** Achint Aggarwal, Frank Kirchner

**Affiliations:** 1 DFKI GmbH, Robotics Innovation Center (RIC), Robert-Hooke-Str. 1, Bremen D-28359, Germany; E-Mail: frank.kirchner@dfki.de; 2 Robotics Group, Department of Mathematics and Computer Science, University of Bremen, Robert-Hooke-Str. 1, Bremen D-28359, Germany

**Keywords:** haptic object recognition, tactile sensor, database matching, object exploration, biological exploration, particle filter, edge following, tactile images, recognition by parts, underwater recognition

## Abstract

Tactile sensors, because of their intrinsic insensitivity to lighting conditions and water turbidity, provide promising opportunities for augmenting the capabilities of vision sensors in applications involving object recognition and localization. This paper presents two approaches for haptic object recognition and localization for ground and underwater environments. The first approach called Batch Ransac and Iterative Closest Point augmented Particle Filter (BRICPPF) is based on an innovative combination of particle filters, Iterative-Closest-Point algorithm, and a feature-based Random Sampling and Consensus (RANSAC) algorithm for database matching. It can handle a large database of 3D-objects of complex shapes and performs a complete six-degree-of-freedom localization of static objects. The algorithms are validated by experimentation in ground and underwater environments using real hardware. To our knowledge this is the first instance of haptic object recognition and localization in underwater environments. The second approach is biologically inspired, and provides a close integration between exploration and recognition. An edge following exploration strategy is developed that receives feedback from the current state of recognition. A recognition by parts approach is developed which uses the BRICPPF for object sub-part recognition. Object exploration is either directed to explore a part until it is successfully recognized, or is directed towards new parts to endorse the current recognition belief. This approach is validated by simulation experiments.

## 1. Introduction

Tactile sensors posses the potential of becoming an indispensable part of a modern day robotic system. While laser and vision sensors have proven their worth in most of the structured and even some unstructured ground based applications, their utility is inhibited by bad lighting conditions. For underwater applications, bad lighting conditions and water turbidity created either by dirt or by sediment stirred by the robot itself, limit the utility of laser and vision sensors. Tactile sensors, on the other hand are immune to ambient lighting conditions and water turbidity because of their intrinsic insensitivity to ambient light. Thus, they are essential for complementing the performance of vision sensors in ground based applications and are capable of performing an even larger role in underwater applications.

However, research in tactile perception remains limited because of the limited availability of tactile sensors, especially for underwater applications. Further, while vision and range sensors have Charge-Coupled Devices (CCD) or Complementary Metal-Oxide-Semiconductor (CMOS) arrays that yield one-shot environment scans, tactile data acquisition is a slow process since data is collected only via direct contact between the tactile sensor and its environment. This is the most important limitation of tactile sensors. Thus, it is desirable to have dense clusters of tactile sensing elements on robot appendages for maximum data acquisition. Also, systematic environment exploration should be planned intelligently and maximum amount of information should be extracted from the acquired tactile data.

The aim of this article is to demonstrate the utility of tactile sensors on a robotic system. We present multiple perspectives and methodologies for haptic object recognition and localization, and prove their utility in ground based and underwater applications. While the focus of the article is on haptic object recognition, we believe that tactile and vision sensors need to work closely to augment the capabilities of each other. The algorithms have therefore been developed keeping the vision interface in mind.

**Figure 1.**
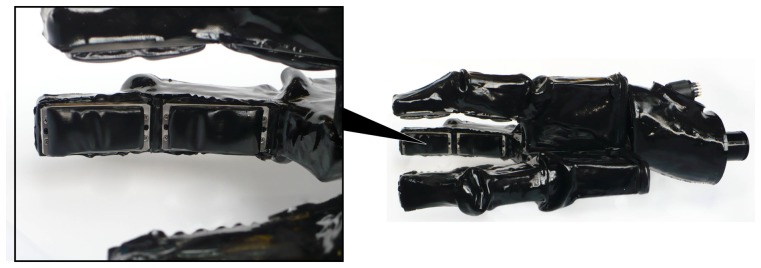
SeeGrip: deep-sea capable gripper with tactile sensing units beneath flexible skin covering.

**Figure 2.**
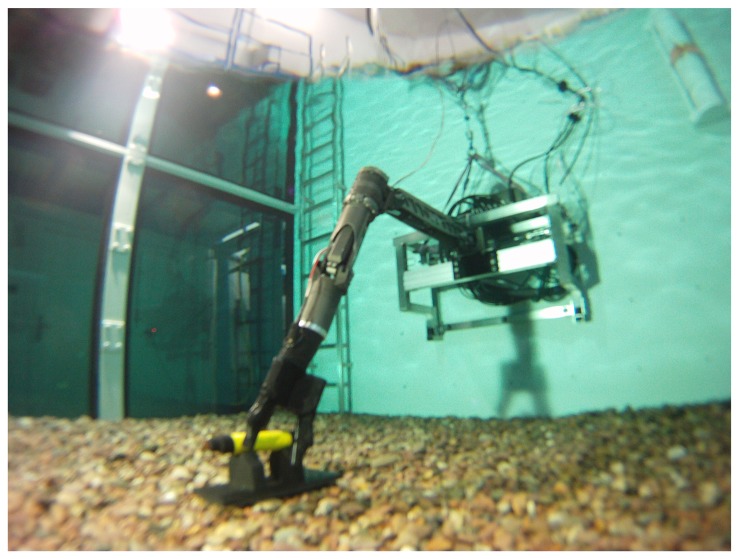
SeeGrip gripper on the Orion7P manipulator in the Underwater Testbed at DFKI GmbH.

**Figure 3.**
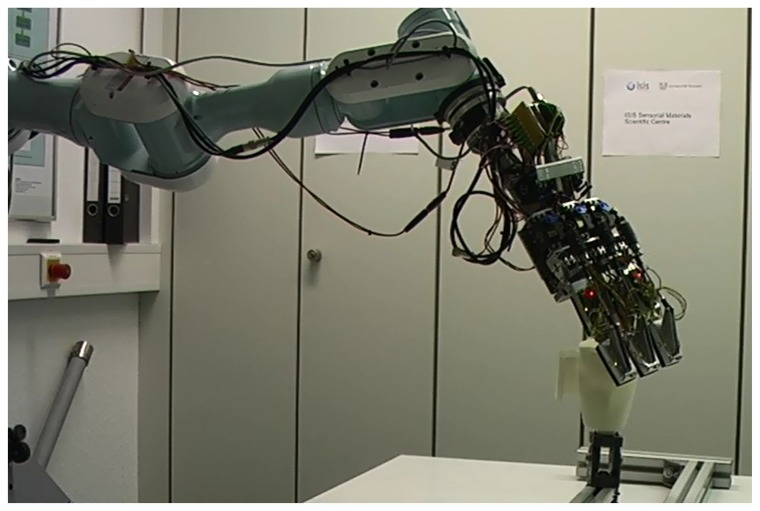
Tactile sensors on a sheet-metal gripper at end-effector (EEF) of a Mitsubishi PA10 manipulator.

Recently, a tactile sensing system with high spatial and force resolutions [1] has been developed which is also capable of underwater use. Water-proof versions of this system have been mounted on the appendages of an underwater gripper called SeeGrip (Figure 1), which is in turn fixed at the end-effector (EEF) of an industrial standard and deep sea capable Orion7P manipulator from Schilling Robotics (Figure 2). Another variant of the tactile sensor has been prepared for ground based applications which is mounted on the fingers of a light weight gripper which is in turn fixed at the EEF of a Mitsubishi PA10 manipulator (Figure 3). While the manipulator and gripper actuators enable the systematic exploration of new and unexplored areas of the object surface, their kinematics and angular encoders enable the fusion of this data collected over several contact iterations into an object map. The high spatial and force resolution of the sensors makes it possible to go beyond a binary contact or no contact detection and enables the estimation of the exact position of contact (Section 3). This precision allows the representation of tactile data as point clouds which opens up the possibility to utilize the state of the art from the laser sensing literature for haptic object recognition.

This article focuses on the problem of tactile sensor based object recognition and complete 6 degree of freedom (dof) localization in structured ground based and underwater environments. We focus on the recognition of static objects, from a pre-known, but large object database. We present two different approaches for haptic object recognition and localization. The first approach called Batch RANSAC and Iterative Closest Point (ICP) augmented Particle Filter (BRICPPF) is based on an innovative combination of particle filters, ICP, and a feature based RANSAC algorithm for database matching.

The representation of tactile data as point clouds allows us to use point cloud matching techniques like RANSAC for database matching [2]. However, the manipulator EEF positioning errors (especially inherent to underwater manipulators [3]) lead to inaccuracies in the object map when data collected from multiple measurements is fused together. This leads to low recognition rates when using point cloud registration alone (as shown by experiments in Section 6.1.4.). From another perspective, the problem of object recognition and localization can be compared to the mobile robot localization problem (Section 4.4). Each database object has its own map. Object recognition is equivalent to determining the correct map and the localization problem refers to finding the position of the tactile sensor with respect to (w.r.t.) the object map. Thus, Bayesian methods like particle filters can be used for tracking and evolving hypotheses over multiple object exploration steps, which has a particular advantage in the case of haptic exploration. Since object exploration is a relatively slow process, the time during which the robot moves can be used for state estimation. Thus, it is advantageous to evolve the state beliefs over time rather than trying to estimate these in one shot using point cloud registration methods. However, particle filters are not efficient in dealing with high dimensional spaces and solving a complete 6 dof localization problem alone could take weeks using generic particle filters.

We therefore use a combination of RANSAC based database matching and particle filters for robust object recognition and localization. The RANSAC based database matching component allows the particles to concentrate only on the high probability regions of the 7 dimensional space and provides an efficient solution to the problems of high dimensions and particle starvation. Sequentially evolving the hypotheses within the particle filter framework allows robustness against noise and leads to convergence to the correct hypothesis within a few measurements only. Using ICP ensures that if an object pose is detected in the vicinity of the correct pose, it will eventually be corrected to the actual pose while evolving with new measurements. This also ensures that if a correct hypothesis was sampled at any time step, it will not be lost after subsequent measurements, which cannot be ensured using point cloud registration alone.

This approach is presented in conjunction with an exploration strategy that diverts attention towards maximum unexplored regions of an object's surface and can be used with grippers of different morphologies. It can handle a large database of 3D objects of complex shapes and performs a complete 6 dof localization of static and non-deformable objects. For practical applications, recognition of pre-known objects from a large database is a reasonable constraint. Further, since it is not practical to create a database from ground truth collected from tactile measurements, especially in underwater applications, we ensure that our methodology deals with a database constructed from laser sensors in the air. This database can be constructed autonomously in simulation and is easily expandable. An approach for incorporating both contact and free space measurements is also presented. Algorithms are validated by experimentation in ground and underwater environments using real hardware. To our knowledge this is the first instance of haptic object recognition and localization in underwater environments.

The second approach is biologically inspired, and is motivated by an efficient integration of the object exploration and object recognition strategies. It is inspired by the current understanding of human haptic perception and other generic human object recognition principles. Some researchers, via several studies [4–7], have shown that humans tend to use a variety of stereotypical hand movement patterns (called exploratory procedures) to measure particular object properties like hardness, texture and shape. Out of these, contour following or moving the fingers around the edge of an object is the most important strategy used for ascertaining the shape and size of an object [5]. It has also been established that the human visual recognition system, in its earliest stages, extracts spatial object information in the form of oriented edges. These edges are combined to produce low level object primitives or features. For example, a set of volumetric primitives called geons have been proposed [8] and it has been suggested that common objects are represented by a spatial combination of these geons. Humans tackle the object recognition problem by comparing an object with representations of various object categories stored in memory.

We therefore use a combination of these biological concepts for developing an efficient exploration and recognition strategy. Our approach is based on exploring an object's sub-part continuously and only until it is satisfactorily recognized. An edge following exploration strategy is developed that concentrates on following the closest lying edge on the object's surface. The tactile data thus collected is used for the recognition of the object's part using the BRICPPF approach discussed above. The exploration module is either directed to explore the part further, or it is directed to explore a new object part depending on the current state of recognition. A recognition by parts approach is developed which spatially fits the identified object parts together to recognize the complete object and determine its 6 dof pose. This approach enables the exploration of only the most informative regions of the object's surface, and thus leads to minimal wastage of tactile data. However, contrary to geons, that are general shape primitives, our approach recognizes specific object sub-parts that are bound to the description of a specific database object. It is shown to work well with objects with well-defined edges and is particularly suited for applications involving only a single tactile sensing unit. An approach for autonomous construction of the database is also presented. The complete exploration and recognition approach is validated via simulation experiments.

This article is structured as follows. Related work is discussed in Section 2. The tactile sensing system is discussed in Section 3. The BRICPPF approach is presented in Section 4, followed by a discussion of the recognition by parts approach in Section 5. Ground based and underwater experiments and results are presented in Section 6. The experimental results and their interpretations are discussed in Section 7 followed by a final conclusion in Section 8.

## 2. Literature Review

Research in tactile sensor based object recognition for underwater applications is rare. However, tactile sensing for ground based object recognition has received a fair amount of attention. Some researchers have concentrated on the reconstruction of the complete shape of unknown objects using tactile sensors [9,10]. This involves the complete exploration of the object surface using an exploration strategy, and definition of the object shape using geometric models like superquadrics. This has been limited to objects with relatively simple shapes, and is an over-kill for the common practical scenario where a known object needs to be recognized quickly using minimal tactile data.

A few researchers have focused on the recognition of pre-known objects by representing the explored object as volumetric models and matching with a known object database [11]. Other researchers [12] use point clouds to represent tactile data and use ICP for object matching. These are however limited to simulation, and their efficiency decreases linearly with the number of objects in the database.

Machine learning approaches have also been used for haptic object recognition. Neural Networks [13], Bag of Features [14] and Self Organizing Maps [15,16] have been used to identify objects in the haptic space (using finger geometry and tactile data) itself without building a complete 3D representation of the object. This involves a complete knowledge of the ground truth of the object, and thus adding additional objects to a database is a complex procedure.

Some methods for active exploration of objects have been proposed [17]. Objects are actively grasped and rolled without slipping between fingers to infer object motion and thus fuse the tactile data gathered at different times. This is however, relevant only to portable objects, objects smaller than the gripper, convex objects with smooth surfaces and known friction properties, and the availability of hardware and controllers capable of such operations.

Bayesian techniques for tactile sensor based localization have also been previously reported. One recent approach [18] presents the application of particle filters and histogram filters for 3 dof localization of 2D objects using a dense array of tactile sensors, similar to the sensors used in the present article. Occupancy maps of objects built with the real sensor data are used as a database. These occupancy maps are queried with tactile sensor measurements to update the probabilities of the beliefs. The approach is shown to deal with a database of five 2D objects which are assumed to be static. Another approach [19] presents Bayesian methods for fast localization of an object in complete 6 dof space using just a single tactile probe. The method uses annealing with a particle filter and gradually scales precision from a coarse to fine level. This provides a solution to the large dimensionality problem, but the approach cannot recover if it loses track of the correct state of the object. Also, the approach is presented for the localization of an object of known identity. It uses a polygonal representation of the object to estimate planar surfaces from contact which becomes complicated for objects of complex shapes. The approach is also shown to work when Gaussian noise is used to model the movement of a non-static object which is similar to our case of large EEF positioning errors inherent to underwater manipulators.

A few researchers have discussed the exploration strategies required for object exploration. A probabilistic task-based approach has been presented [20] for selection of the next best exploration direction that allows the achievement of the desired grasps. A state estimator keeps track of the current state of an object and an action selector selects the most appropriate action that maximizes the likelihood of success. The action domain consists of a pre-defined set of action sequences and a look-ahead procedure is used for the action selection. Several approximations are presented for efficiently managing the branching for this look-ahead procedure for 3-dof localization of a known object. [21] presents an information gain approach for selecting the next best exploration direction. This strategy is based on minimizing the entropy of the belief distribution. The approach is proven to work well for localization of a known object. The application of both these approaches to recognition from a large database and subsequent 6 dof localization would need several modifications for managing the large search space. Other approaches [22,23] present single finger tactile exploration strategies for recognizing polyhedral objects. Some others [24,25] have investigated the procedures for haptic exploration of object features. Recently, some approaches [26,27] have been proposed to direct exploration towards the maximum unexplored zones. Another strategy [12] is adaptable to specific feature exploration or to exploration of unexplored zones. Both our exploration strategies (Sections 4.1.3. and 5.1) do not require knowing the identity of the object being explored and can generate exploration trajectories on the fly without needing a pre-defined action set. Further, our widest unexplored cone exploration strategy (Section 4.1.3.) can deal with objects of complicated shapes as well.

An article [28] provides an excellent review of the state of the art in object recognition techniques from range sensing applications. Random Sampling and Consensus (RANSAC) [29] is the most widely used technique for registration of point cloud data. The literature consists of methods for local surface feature based matching [30–32] where the sensor data is rich enough for reliable local surface feature estimation. At the other end of the spectrum are global shape matching algorithms [33,34] that are relevant to applications where sufficient information about the global shape of objects is available. Finally, there are methods [35] that combine the local features and global shape estimation to recognize database objects. A local feature based database pruning approach [36] allows efficient query of large object databases.

## 3. Hardware

SeeGrip (Figure 1) is a three-fingered deep-sea capable hydraulic gripper with multimodal sensing functionality and has been designed to work under ambient pressure of at least 600 bar which corresponds to a depth of 6 km under water. Detailed information regarding the hydraulic finger actuation is provided in a recent publication [37]. Information regarding the various sensing modalities, pressure tolerant electronics for deep-sea operations, and the low level processing architecture can be found in some other publications [1,38].

In this section, we will discuss the characteristics of the tactile sensing modality based on the fiber optic measurement principle. This modality is used for detecting geometric properties of the objects in contact and is the most relevant sensing modality for this article. Thus, its working principle is also presented briefly for completion.

**Figure 4.**
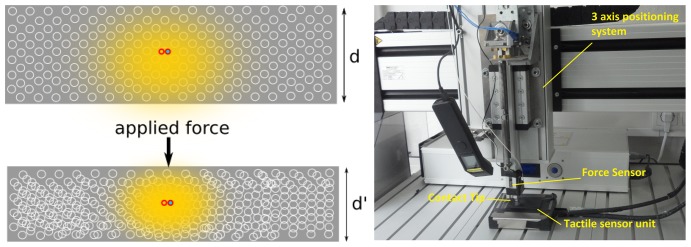
(**Left**) Working principle of the fiber optic sensor [39]. (**Right**) Metaksa Testbench for testing sensor characteristics.

The working principle of the fiber optic sensors is depicted in Figure 4 (left). Each sensor element (sensel) consists of two optical fibers and a foam material. The endings of the optical fibers are placed within the foam or directly in front of it. One of the optical fibers leads light into the cell structure of the foam. The second optical fiber works as a brightness sensor. The scattered light in the foam structure is sensed and transferred to a photo-transistor. Application of force to the foam structure leads to a compression of the foam which results in a change in the scattering of the emitted light. This change is sensed and transferred to the photo-transistor. The intensity of the sensed light is directly proportional to the amount of compression of the foam in front of the fiber optic element.

For hardware experiments described in this article, sensor modules consisting of an array of 72 sensel elements with a spatial resolution of 4 mm have been used. The sensors have a base size of 6 by 3 cm, with the foam thickness being 4 mm. For simulation experiments for edge following, a high resolution version consisting of an array of 324 sensel elements with a base size of 6 by 3 cm has been used. The hardware for this version is currently under development. Since most tactile sensors vary in their sensitivity from sensel to sensel, a calibration routine has been developed to map the amount of foam compression to the observed output for each sensel. Thus, each sensel is scaled individually and can output the amount of compression of the part of the foam directly in front of it. Finally, force-torque sensors at the base of each finger sense the absolute applied forces on each finger and are used to detect contact with the finger at a basic level.

**Figure 5.**
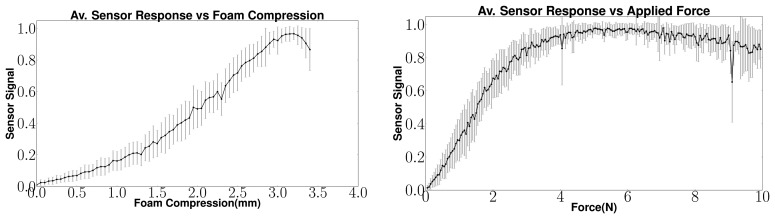
Mean sensor response (with standard deviation) of all sensels to foam compression (**left**) and applied force (**right**).

For observing the signal response, a fiber optic sensor with 72 sensel elements was tested on a Metaksa Testbench shown in Figure 4. The 3-axis testbench allows the precise positioning of a contact tip on the sensor unit such that each sensel can be compressed by the desired amount. A force sensor monitors the applied contact force. A 5 mm by 5 mm tip was used to gradually compress each individual sensel one by one. The response of the sensels was recorded along with the amount of foam compression and the applied force. The mean sensel response for all sensels (with standard deviation) is shown in Figure 5 w.r.t. the foam compression and the applied force. It can be observed that the sensor signal increases steadily with increasing foam compression. However, after about 80% of the foam compression (total thickness 4 mm), the sensor response drops. This is because the intensity of light reflected in the foam starts decreasing after the foam has been compressed beyond 80% of its thickness. This plot shows that the sensel signals can be directly used to interpret the amount of foam compression. The corresponding sensor response *versus* the applied force is also shown for clarity.

For under water operation, the foam is filled with translucent silicone oil which also fills the free spaces between the internal gripper components and its outer skin. The application of external force to the sensor squeezes the silicone oil out of the foam and removal of the force leads to retraction of the foam and seepage of silicone oil back into the foam structure. The necessity of using silicone oil within the sensor setup leads to some changes in the behavior of the measurement principle as compared to the air filled foam. Since the sensor principle is based on the scattering of light in the foam structure, using silicone oil in the foam rather than air results in a dampening of the light feedback on the photo-transistor. With a silicone filled foam with 4 mm thickness, compression changes below half a millimeter can be sensed.

## 4. BRICPPF for Object Recognition and Localization

This section presents the BRICPPF object recognition and localization methodology. The object exploration strategy, sensor model and data fusion methods are discussed first. An autonomous object database creation methodology is presented in Section 4.2. The local feature and global shape based database matching methodology is discussed in Section 4.3. Finally, the particle filter formulation and recognition methodology is discussed in Section 4.4.

### 4.1. Object Exploration for Tactile Data Collection

This section discusses a sensor model that has been developed for estimating the point of contact in the local tactile sensor frame. This is converted to global representation using the forward kinematics of the robot system. Free space is evaluated using the robot swept volume and an object representation is created by fusing multiple measurements as discussed below in Section 4.1.2. An exploration strategy uses this representation to divert attention to maximum unexplored zones as discussed below in Section 4.1.3.

#### 4.1.1. Sensor Model

Tactile data is transmitted from the SeeGrip gripper to an external computer in the form of pre-processed tactile images. A tactile image contains the signal value for each sensel of a fiber optic sensor array. Additionally, it also carries the absolute angular positions of all gripper joints at the time the tactile data was recorded. As stated in the previous section, our sensors can be used to determine the exact position of contact rather than just a binary contact or no contact estimation.

Let *e_j_* denote the signal value for a sensel element *j* ∈ [1, *n*] where *n* is the number of sensel elements in a sensor array. A threshold of 10% of the maximum expected signal *e_max_* is used to filter out the non contacting sensels. For the sensels on which contact is detected (*e_j_* > 0.1 × *e_max_*), linear interpolation on *e_j_* is used for estimating the compression of the foam, and subsequently the height of contact on the sensel $z_{j}^{*}$.

(1) $$z_{j}^{*}=z_{\text{max}}\frac{\left(e_{\text{max}}-e_{j}\right)}{e_{\text{max}}}$$

Assuming a Gaussian sensor noise with mean $z_{j}^{*}$ and standard deviation *σ_sensel_*, the final height of contact on the sensel *z_j_* is sampled from the normal distribution 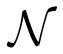 (*z_j_*, $z_{j}^{*}$, $\sigma_{\text{sensel}}^{2}$).

(2) $$\text{N}(z_{j},z_{j}^{*},\sigma_{\text{sensel}}^{2})=\frac{1}{\sqrt{2\pi \sigma_{\text{sensel}}^{2}}}e^{-\frac{1}{2}\frac{{\left(z_{j}-z_{j}^{*}\right)}^{2}}{\sigma_{\text{sensel}}^{2}}}$$

Since the spatial position (*x_j_*, *y_j_*)*^L^* of sensel *j* w.r.t. the local sensor frame is pre-known from the Computer-Aided Design (CAD) construction models, (*x_j_*, *y_j_*, *z_j_*)*^L^* represents the complete position of a contact point in the local sensor frame.

#### 4.1.2. Data Fusion: Contact and Free Space

The tactile data from all the sensors at the end of each grasping sequence (grasping sequences are discussed in the next section) is transmitted to an external computer. The sensor model discussed above is used to determine the contact positions $P_{i}^{L}={\left(x_{i},y_{i},z_{i}\right)}^{L}$, *i* ∈ [1, *N*] in the local sensor frames, where N is the total number of contact points. The forward kinematics of the gripper is used to transform these local positions $P_{i}^{L}$ to positions ( $P_{i}^{W}$) in a fixed world coordinate frame using the following equation:

(3) $$P_{i}^{W}=T_{\text{EEF}}(\Theta_{\text{arm}})T_{\text{sensor}}(\Theta_{\text{finger}})P_{i}^{L}$$

In Equation (3)*T_EEF_*(Θ*_arm_*) is the homogeneous transformation defining the forward kinematics of the robot arm upto its EEF. *T_sensor_*(Θ*_finger_*) is the homogeneous transformation of the sensor containing sensel *i* w.r.t. the EEF and defines the the forward kinematics of the corresponding gripper finger.

All contact points $P_{i,t}^{W}$ collected at a time instance *t* are added together to form a point cloud $PC_{t}^{\text{cont}}$. To maintain a cumulative representation of the explored object's surface, a cumulative point cloud $PC_{\text{cum},t}^{\text{cont}}$ is maintained that consists of all measurements collected till time *t*. To add $PC_{t}^{\text{cont}}$ to $PC_{\text{cum},t-1}^{\text{cont}}$, all points in $PC_{t}^{\text{cont}}$ are simply appended to $PC_{\text{cum},t-1}^{\text{cont}}$ and a Voxel grid filter [40] is used to filter out overlapping or very closely spaced points.

For estimating the free-space, a 3D occupancy map [41] of the explored region is maintained. At each exploration step, this map is updated with contact point positions $PC_{t}^{\text{cont}}$. The swept volume of the gripper links is used as free space to update the occupancy map. The occupancy grid is maintained in an Octree representation for efficient update and search operations.

At any time *t*, the free space can be represented as a free space point cloud $PC_{t}^{\text{free}}$. This is done by querying the occupancy map for free space Octree leaves within a bounding box *B* around the explored object. The size of the bounding box {*B_x_*, *B_y_*, *B_z_*} is defined by the maximum expected length of an object *s_objmax_* along any dimension in the complete object database (see Section 4.2). At any time, a small bounding box *B^cont^* with dimensions { $B_{x}^{\text{cont}}$, $B_{y}^{\text{cont}}$, $B_{z}^{\text{cont}}$} is determined that completely contains all points in $PC_{\text{cum},t}^{\text{cont}}$. Then,

(4a) $$B_{x}=B_{x}^{\text{cont}}+2s_{{\text{obj}}_{\text{max}}}$$

(4b) $$B_{y}=B_{y}^{\text{cont}}+2s_{{\text{obj}}_{\text{max}}}$$

(4c) $$B_{z}=B_{z}^{\text{cont}}+2s_{{\text{obj}}_{\text{max}}}$$

Equation (4) ensures that for any possible object match hypothesis that contains at least one point from $PC_{\text{cum},t-1}^{\text{cont}}$, no surface contact point is possible outside the box *B* for all database objects. The center of *B* is defined by the centroid of $PC_{\text{cum},t}^{\text{cont}}$. To generate $PC_{t}^{\text{free}}$, the free space Octree leaves are sampled at a fixed resolution *res_free_*, such that only one point lies inside a cube of size *res_free_*.

#### 4.1.3. Exploration of Widest Unexplored Zones

An exploration strategy that focuses attention towards widest unexplored regions on the object surface has been developed. Motion planning for the manipulator and gripper systems is performed as a part of the exploration strategy. The exploration strategy is broadly divided into two parts as follows.

**Figure 6.**
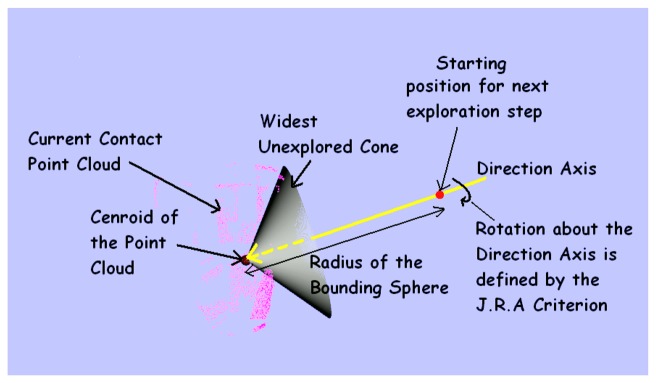
Widest Unexplored Cone based coarse exploration strategy.

Firstly, a *Coarse Exploration* strategy is responsible for the motion planning of the robot arm to which the gripper is attached (see Figures 2 and 3). At time step *t*, the maximum unexplored region on the object surface is determined by the widest open cone formed by the contact points in $PC_{\text{cum},t}^{\text{cont}}$, with its vertex at the centroid of $PC_{\text{cum},t}^{\text{cont}}$ (see Figure 6). The bounding sphere of the object is defined by a sphere of radius twice the maximum object size *s_obj_max__* with its center at the centroid of $PC_{\text{cum},t}^{\text{cont}}$. The next exploration direction (for the normal vector of the middle finger's proximal tactile sensor) is defined by the axis of the cone, and the position of the wrist is given by the point where this axis intersects the bounding sphere. The direction and 3D position define 5 parameters for the wrist pose. The complete 6 dof pose of the wrist is determined by using the Joint Range Availability redundancy resolution technique [42]. Assuming the object to be in an obstacle free environment with a known position of the floor, collision free paths for the manipulator are planned from starting to the end configuration using *Rapidly Exploring Random Trees* based path planners [43] and by assuming the bounding sphere to be occupied. Finally, the wrist is moved straight in the new exploration direction till there is contact with the object (detected by force-torque sensors).

Secondly, a *Fine Exploration* strategy is responsible for maximizing the exploration of the object from a fixed wrist position using all the degrees of freedom of the gripper. Actions like *Close Fingers* and *Open Fingers* are used for fine exploration. The execution of the grasping behaviors is monitored by the controllers inside the SeeGrip hand. The hand uses feedback from the force torque sensors and tactile sensors to determine when a grasping behavior has been completed. Tactile images are only transmitted to the external computer (for measurement update in Section 4.4.3.) at instances when the *Close Fingers* grasping behavior is completed.

### 4.2. Object Database

Since most underwater and ground based robotic applications require the recognition of *known* objects, having a pre-knowledge of the expected objects in the environment is advantageous. The database consists of objects in the form of their surface 3D position point clouds of two different resolutions: fine and coarse. Each point of the coarse point cloud also carries a feature description which encodes the characteristics of the local shape of the surface at that position. To maintain generality, two necessary requirements have been ensured. Firstly, the database can be created autonomously and new objects can be easily added. Secondly, it is not necessary to have the object database created by the exact ground truth collected via real tactile sensors. These two points distinguish this research from other approaches [15,16,18] where ground truth is required for creating the training data set.

**Figure 7.**
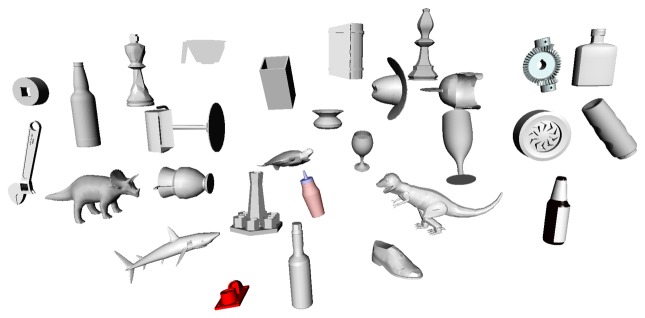
A few objects from the database of 45 objects.

The database is created in simulation and requires a CAD model of the object. A simulated laser scanner is used to scan the object surface completely. For autonomous exploration of the complete object, the widest cone exploration strategy (Section 4.1.3.) is used to position the laser scanner to divert attention to maximum unexplored zones. For escaping local minima, random directions are used after a fixed number of iterations. The complete object surface contact cloud is computed by combining the contact point clouds generated by the laser scanner and filtering with a voxel grid to eliminate overlapping points. Finally, the features (Section 4.3.1.) of this contact cloud are calculated. The object database consists of 45 objects (Figure 7), most of them from the Princeton Shape Benchmark Database [44], with similar sizes (maximum length of 20 cm) such that they can be grasped by the SeeGrip gripper.

### 4.3. Local Features and Global Shape Matching

For fast and efficient database matching, a local feature and global shape matching approach is employed. This algorithm combines feature-based model indexing for initial pruning of large databases (similar to [36]) and geometric constraint based alignment (with RANSAC) using only the pruned database for efficient object recognition.

#### 4.3.1. Feature Selection

A variety of features and signatures presented in literature were compared for selecting the most appropriate feature for our application. The complete comparison procedure is presented in Appendix A. Fast Point Feature Histograms [45] was chosen as the most appropriate feature for the tactile sensing application.

#### 4.3.2. Batch RANSAC Algorithm for Database Matching

**Initialization:** For each database object *obj*, the contact point cloud $PC_{db,\text{obj}}^{\text{cont}}$, its k-d tree [46] $KT_{db,\text{obj}}^{\text{cont}}$, and feature point cloud $PC_{db,\text{obj}}^{\text{feat}}$ are loaded. $PC_{db,\text{obj}}^{\text{feat}}$ for all objects are together loaded into a Cumulative feature k-d tree $KT_{db,\text{cum}}^{\text{feat}}$. At any time *t* during object exploration, the exploration contact point cloud $PC_{\text{cum},t}^{\text{cont}}$ (Section 4.1.2.) can be matched with the database.

**Triplet sampling:** Three points (*p*_1_, *p*_2_, *p*_3_) are randomly sampled from $PC_{\text{cum},t}^{\text{cont}}$ such that they are spaced away from each other (using a predefined minimum distance threshold) and do not lie on the boundaries of the point cloud patches (to facilitate reliable feature calculation). The features (*f*_1_, *f*_2_, *f*_3_) for these three points are calculated.

**Feature based database pruning:** For each feature *f*_1_, *f*_2_ and *f*_3_, the closest matching *W* features are determined by querying $KT_{db,\text{cum}}^{\text{feat}}$. They are collected in three sets *F_db_*_,1_, *F_db_*_,2_ and *F_db_*_,3_ which forms the new reduced search space for registration.

**Triplet registration**: One feature point is randomly sampled from each of *F_db_*_,1_, *F_db_*_,2_ and *F_db_*_,3_, such that all three points belong to the same database object (say *obj*) and their corresponding points *p_obj_*_,1_, *p_obj_*_,2_ and *p_obj_*_,3_ in $PC_{db,\text{obj}}^{\text{cont}}$ have similar inter-point spatial distances as the input points *p*_1_, *p*_2_ and *p*_3_. Singular Value Decomposition [47] between (*p_obj_*_,1_, *p_obj_*_,2_, *p_obj_*_,3_) and (*p*_1_, *p*_2_, *p*_3_) is used to calculate the 6 dof transformation *T* = {*pos*, *orient*} between $PC_{db,\text{obj}}^{\text{cont}}$ and $PC_{\text{cum},t}^{\text{cont}}$.

**Match evaluation**: To evaluate the match, $PC_{\text{cum},t}^{\text{cont}}$ is transformed by *T*^−1^ to the coordinate frame of the database object (*obj*). This allows efficient error estimation using $KT_{db,\text{obj}}^{\text{cont}}$. For each point *c_i_*, *i* ∈ [1, *C*] in the transformed $PC_{\text{cum},t}^{\text{cont}}$, the distance *d_ci_* to the closest neighboring point in $PC_{db,\text{obj}}^{\text{cont}}$ is evaluated using $KT_{db,\text{obj}}^{\text{cont}}$. The error contribution of each point *c_i_* is given by:

(5) $$e_{c_{i}}^{\text{cont}}=\{\begin{array}{ll} {\left(\frac{d_{c_{i}}}{d_{\text{outlier}}}\right)}^{2} & \text{if}\;d_{c_{i}}<d_{\text{outlier}}\\ 1 & \text{if}\;d_{c_{i}}\geq d_{\text{outlier}} \end{array}$$

where *d_outlier_* is the outlier distance threshold.

The complete weight *wt* of the match is determined by:

(6) $$wt=\prod_{i=1}^{C}\text{exp}(-\frac{1}{C}e_{c_{i}}^{\text{cont}})$$

For each sampled triplet from the input, the *Triplet registration* step is repeated *R* times to generate *R* object and pose hypotheses *S* = {*s*_0_, *s*_1_, …, *s_R_*} where *s_i_* = {*obj_i_*, *pos_i_*, *orient_i_*, *wt_i_*}, *i* ∈ [1, *R*]*. pos_i_* and *orient_i_* represent the 3D position and orientation respectively.

**Output**: *Triplet sampling* and all subsequent steps are repeated *N* times to yield *N* * *R* weighed hypotheses. The hypothesis with the maximum weight is considered as the best match. The corresponding *obj* is considered as the recognized object and the 6 dof pose {*pos*, *orient*} is considered as the recognized object's pose.

### 4.4. Sequential State Estimation with BRICPPF

In this section, a sequential state estimation approach is presented that is built upon the Batch RANSAC approach presented above, but enables robust object recognition and pose estimation after only a few exploration steps. This is achieved by keeping track of the top hypotheses from each Batch RANSAC output and successively evolving them with new measurements from each exploration step. These hypotheses represent the most promising parts of the search space and thus more computational power is diverted to these regions.

#### 4.4.1. Problem Formulation

To describe the particle filter formulation, we use the terminology from [48] and [18]. The object recognition and localization task is considered as a sequential state estimation problem, where, at each time step *t*, the tactile sensor measurements are given by *z_t_*, and the sensor state *x_t_* has to be determined at which the most recent measurement (*z_t_*) was taken. The state *x_t_* = {*obj*, *pos*, *orient*} represents the object identity *obj* and the complete 6 dof tactile sensor pose $T_{S}^{O}=\{\text{pos},\text{orient}\}$ in the fixed object coordinate frame. Note that we are eventually interested in the object pose in the fixed robot base frame $T_{O}^{R}$ which can be directly inferred from $T_{S}^{O}$ using $T_{O}^{R}=T_{S}^{R}{\left(T_{S}^{O}\right)}^{-1}$. Here, $T_{S}^{R}$ is the sensor pose in the robot frame and is available from the forward kinematics of the robot (Equation (3)). We assume the object and the robot base frames to be static.

A particle filter [48] is a non-parametric Bayesian filter that can be used to estimate the posterior distribution *p*(*x_t_*∣*u*_1:_*_t_*, *z*_1:_*_t_*, *DB*) over the state at the current time *t* given a series of commands *u*_1:_*_t_* sent to the robot; measurements *z*_1:_*_t_* collected from tactile sensors; and the object database *DB.* The particle filter is a Monte Carlo method where this posterior distribution is represented by set of *n_p_* samples called particles, denoted by $\chi_{t}=\{x_{t}^{\left[1\right]},x_{t}^{\left[2\right]}\ldots x_{t}^{\left[n_{p}\right]}\}$ with an importance weight $w_{t}^{\left[i\right]}$, *i* ∈ [1, *n_p_*] associated with each sample. At each time step, a motion update and a measurement update is performed.

#### 4.4.2. Motion Update

The motion update requires the estimation of *p*(*x_t_*∣*x_t_*_−1_, *u_t_*) for a control command *u_t_*. For each particle, the control command effects $T_{S}^{R}$ which is known from the forward kinematics of the robot and it does not change *obj.* Because of the noise in the estimation of $T_{S}^{R}$ for industrial underwater manipulators, the components of the particle's pose are artificially corrupted with Gaussian noise. For estimating manipulator EEF pose uncertainty for a particle $x_{t}^{\left[k\right]}=\{{\text{obj}}_{t}^{\left[k\right]},{\text{pos}}_{t}^{\left[k\right]},{\text{orient}}_{t}^{\left[k\right]}\}$ we use noise sampled from 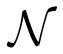
(0**,** *σ*) with *σ* = 1 cm for each axis of ${\text{pos}}_{t}^{\left[k\right]}$ and *σ* = 5 degree for each axis of ${\text{orient}}_{t}^{\left[k\right]}$.

#### 4.4.3. Measurement Update

The purpose of the measurement update is to assign an importance weight to a particle. This weight is computed from a measurement likelihood model *p*(*z_t_*∣*x_t_*, *DB*). Both the contact point cloud and free space point cloud are used to estimate *p*(*z_t_*∣*x_t_*, *DB*) as given by the following equation (Note that contributions from other sensing modalities like hardness, texture, temperature and even vision and laser sensors can also be added to this equation.)

(7) $$p(z_{t}|x_{t},DB)=p_{\text{cont}}(z_{t}|x_{t},DB)*p_{\text{free}}(z_{t}|x_{t},DB)$$

In Equation (7), *p_cont_*(*z_t_*∣*x_t_*, *DB*) is the likelihood from contact measurements given by:

(8) $$p_{\text{cont}}(z_{t}|x_{t},DB)=\prod_{i=1}^{C}\text{exp}(-\frac{1}{C}e_{c_{i}}^{\text{cont}})$$

where $e_{c_{i}}^{\text{cont}}$ is the contact error associated with each point *c_i_*, *i* ∈ [1, *C*] in $PC_{t}^{\text{cont}}$ and given in Equation (5).

For free space contribution, $PC_{t}^{\text{free}}$ (containing Q points) is transformed by ${\left(T_{O}^{R}\right)}^{-1}$ corresponding to the particle. Then for each point *q_i_*, *i* ∈ [1, *Q*] in transformed $PC_{t}^{\text{free}}$, the distance *d_free_i__* to the closest existing point in $PC_{db,\text{obj}}^{\text{cont}}$ is calculated using $KT_{db,\text{obj}}^{\text{cont}}$. The free space error contribution of each point *q_i_* increases with reducing distance *d_free_i__*, and is given by:

(9) $$e_{q_{i}}^{\text{free}}=\{\begin{array}{ll} \frac{d_{\text{thresh}}^{2}-{d_{{\text{free}}_{i}}}^{2}}{d_{\text{thresh}}^{2}} & \text{if}\;d_{{\text{free}}_{i}}\leq d_{\text{thresh}}\\ 0 & \text{if}\;d_{{\text{free}}_{i}}>d_{\text{thresh}} \end{array}$$

where *d_thresh_* denotes the maximum distance threshold for *d_free_i__*, beyond which the free space point has no error contribution. This depends on the filter size used for estimating the free space cloud (Section 4.1.2.) and is given by $d_{\text{thresh}}=\frac{1}{2}res_{\text{free}}$.

Thus, the second term *p_free_*(*z_t_*∣*x_t_*, *DB*) in Equation (7) (the likelihood from free space measurements) is given by:

(10) $$p_{\text{free}}(z_{t}|x_{t},DB)=\prod_{i=1}^{Q}\text{exp}(-\lambda e_{q_{i}}^{\text{free}})$$

where λ is a parameter used for adjusting the relative contribution of free space and contact space.

#### 4.4.4. BRICPPF Algorithm

**Initialization**: Using the initial contact point cloud, $PC_{\text{cum},0}^{\text{cont}}$ and free space cloud $PC_{0}^{\text{free}}$, the Batch RANSAC algorithm (Section 4.3.2.) generates an initial State $\chi_{0}=\{x_{0}^{\left[1\right]}\ldots x_{0}^{\left[n_{p}\right]}\}$, consisting of weighed object and pose hypotheses < $x_{t}^{\left[i\right]}$, $w_{t}^{\left[i\right]}$ > *i* ∈ [1, *n_p_*] (Section 4.4.1.).



**Algorithm 1** BRICPPF Algorithm.
1:**procedure** ParticleFilter(*χ_t_*_−1_, *u_t_*, *z_t_*, 
$PC_{\text{cum},t}^{\text{cont}}$, *DB*)2: *χ̄_t_* = *χ_t_* = Φ3: **for**
*k* = 1 to *n_p_*
**do**4:  Sample 
${\bar{x}}_{t}^{\left[k\right]}∼p(x_{t}|u_{t},x_{t-1}^{\left[k\right]})$5:  
$x_{t}^{\left[k\right]}=\text{ICP}({\bar{x}}_{t}^{\left[k\right]},PC_{\text{cum},t}^{\text{cont}},DB)$6:  
$w_{t}^{\left[k\right]}=p(z_{t}|x_{t}^{\left[k\right]})$7:  
${\bar{\chi }}_{t}={\bar{\chi }}_{t}+<x_{t}^{\left[k\right]},w_{t}^{\left[k\right]}>$8: **for**
*k* = 1 to Ψ * *n_p_*
**do**9:  Draw *i* with probability *α*
$w_{t}^{\left[i\right]}$10:  Add 
$x_{t}^{\left[i\right]}$ to *χ_t_*11: 
$<\chi_{t}^{\prime },{\text{W}}_{t}^{\prime }>=\{<x_{t}^{{\left[1\right]}^{\prime }},w_{t}^{{\left[1\right]}^{\prime }}>\ldots .<x_{t}^{{\left[N\right]}^{\prime }},w_{t}^{{\left[N\right]}^{\prime }}>\}=\text{BatchRansac}(PC_{\text{cum},t}^{\text{cont}},DB)$12: **for**
*k* = 1 to (1 − Ψ) * *n_p_*
**do**13:  Draw *i* with probability *α*
$w_{t}^{{\left[i\right]}^{\prime }}$14:  Add 
$x_{t}^{{\left[i\right]}^{\prime }}$ to *χ_t_*15: **for**
*k* = 1 to *n_p_*
**do**16:  
$w_{t}^{\left[k\right]}=\frac{1}{n_{p}}$


**Motion and Measurement Updates:** The ICP augmented particle filter algorithm is shown in Algorithm 1. The inputs for this algorithm are the particle set *χ_t_*_−1_, the control input *u_t_*, measurement *z_t_*, cumulative contact point cloud $PC_{\text{cum},t}^{\text{cont}}$ and the database *DB.* The algorithm first constructs a temporary particle set *χ̄_t_* which represents the prediction belief $\bar{\text{bel}}(x_{t})$ [48]. This is eventually transformed to the set *χ_t_* which represents the posterior distribution *bel*(*x_t_*). Line 4 shows the motion update on each particle $x_{t-1}^{\left[k\right]}$ in *χ_t_*_−1_ to generate a temporary set of particles ${\bar{x}}_{t}^{\left[k\right]}$, *k* ∈ [1, *n_p_*]. In line 5, for each particle in this temporary set, ICP is performed between $PC_{\text{cum},t}^{\text{cont}}$ and the $PC_{db,\text{obj}}^{\text{cont}}$ transformed to the pose represented by the particle. Here, *obj* is the database object identity represented by this particle. This results in a new particle $x_{t}^{\left[k\right]}$ in the vicinity of ${\bar{x}}_{t}^{\left[k\right]}$ with its pose being a better representation of the new measurement (explained below). Line 6 calculates the importance factor for each particle using the measurement update (Section 4.4.3.).

**Re-Sampling:** In lines 8–10, Ψ * *n_p_* particles are sampled without replacement from *χ̄_t_* to form *χ_t_*. In line 11, a new set of weighed particles is generated from the Batch RANSAC algorithm (Section 4.3). The remaining (1 − Ψ) * *n_p_* particles are sampled from this set, proportional to their weights and added to *χ_t_* as shown in lines 12–14. The weights are normalized in lines 15,16. The final set *χ_t_* represents the posterior distribution *bel*(*x_t_*).

At a given step *t,* the hypothesis $x_{t}^{\left[k\right]}$ in *χ_t_* with the maximum weight evaluated by the product of cumulative contact cloud weight $\prod_{i=1}^{C}\text{exp}(-\frac{1}{C}e_{c_{i}}^{\text{count}})$ of Equation (6) and free space weight $\prod_{i=1}^{Q}\text{exp}(-\lambda e_{q_{i}}^{\text{free}})$ of Equation (10) represents the recognized object ${\text{obj}}_{t}^{k}$ and its 6 dof pose { $pos_{t}^{\left[k\right]}$, ${\text{orient}}_{t}^{\left[k\right]}$}.

**Role of ICP:** ICP based particle pose correction in line 5 can be intuitively explained by assuming that a particle represents a blown up region in the vicinity of the 6 dof pose that it represents. The region represented by a particle is not necessarily unique and could overlap with another particle's region. The ICP step finely modifies (improves) the pose of the original particle by incorporating the latest tactile sensor measurement. By putting a limit on the maximum allowed change in pose with ICP, we ensure that the new particle still remains in the vicinity of the original particle. This pose correction operation with ICP allows us to efficiently tackle the problem of dimensionality with particle filters. This is also based on the fact that even if we considered a cluster of particles in this blown up region, only one particular particle (closest to the correct object pose) would get the highest weight after the measurement update. Thus, tracking only one particle in a region is sufficient with the assumption that ICP would subsequently detect the best pose in this region. Thus, instead of wasting computation effort on multiple particles in a region, we focus on tracking multiple high-probability hypotheses which are spread around the 7D search space, especially in the initial stages of exploration.

## 5. Edge Following and Recognition by Parts

This section presents the edge following based exploration and sub-part fitting based recognition strategies. The exploration strategy is discussed first followed by a discussion of the object database and its autonomous creation in Section 5.2. The architecture that integrates exploration and recognition along with the data flow is introduced in Section 5.3 followed by the object recognition algorithms and feedback mechanisms.

### 5.1. Exploration Strategy

The basic motivation of the complete approach is to concentrate on exploring a part of an object until it is satisfactorily recognized. This imposes a constraint that the exploration strategy should be able to incorporate feedback from the recognition module and also function independently. Thus an edge following exploration strategy has been used which allows the object exploration to function independent of the recognition module and also allows the incorporation of feedback (as will be shown below). Edge following has also been proven to be the most important exploration procedure used by humans for object shape recognition [5]. Further, since edges represent distinct regions on an object's surface, they can be used to segment object sub-parts. The procedure is explained below.

#### 5.1.1. Edge Detection

**Figure 8.**
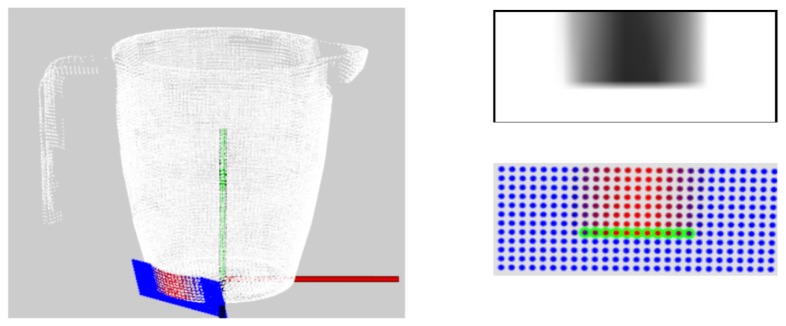
Tactile image processing for edge detection. The left image shows the position of contact between a tactile sensor an object. The gray-scale tactile image is shown in the top right figure and the extracted edge points are shown in lower right figure. The lower right figure also shows all the 324 sensels in the tactile sensor array.

We use the notion of tactile images for segmenting edges in tactile sensor measurements. When a tactile sensor- consisting of a dense distribution of sensels- contacts an object surface, the resulting measurements can be considered as a gray-scale tactile image. Each pixel of the image corresponds to a particular sensel and its gray-scale value corresponds to the amount of compression of this sensel as shown in Figure 8. Then, a combination of edge detection algorithms like Canny [49], and Sobel [50] filters are used for segmenting edges from the image. The points of contact of the sensels corresponding to the edge are determined using Equations (1) and (2). These are converted to 3D points using the forward kinematics of the robot (Equation (3)).

#### 5.1.2. Edge Following

A global edge representation is maintained where each cumulative global edge that is not connected to any other edge is independently tracked. When a new edge is detected in a tactile image, its 3D points are queried for lying in the vicinity of all existing cumulative edges. If any point lies closer than a preset threshold distance, the new edge is appended to the cumulative edge. If more than one cumulative edges lie within the distance threshold, they are all combined into a single cumulative edge. All end-points for each cumulative edge are also maintained, and a 3D direction is assigned to each end point as follows. For each end-point of a newly created edge in a tactile image, the 2D direction is determined in the plane of the tactile image by using 2D mask filters [50]. These 2D directions are converted to 3D directions using the tactile image transformation determined from the forward kinematics of the robot.

For edge following, the 3D direction associated with the closest lying end-point to the current tactile sensor position is chosen as the next direction for edge following. In case of any conflict, the currently followed cumulative edge is given priority. The sensor is displaced in the next propagation direction by a preset distance. The low level controllers on the gripper are responsible for maintaining contact with the object surface using inputs from the force torque sensors and the tactile sensor itself. If contact with the object is lost, the sensor is moved away from the object in a straight line connecting the center of the sensor to the centroid of the current object point cloud (see Section 4.1). It is then translated in the desired direction and brought back in a straight line towards the object till contact is established again. In this case, the sensor is also rotated about two fixed orthogonal axes lying in the plane of the sensor for maximizing contact with the object surface.

### 5.2. Object Database

**Figure 9.**
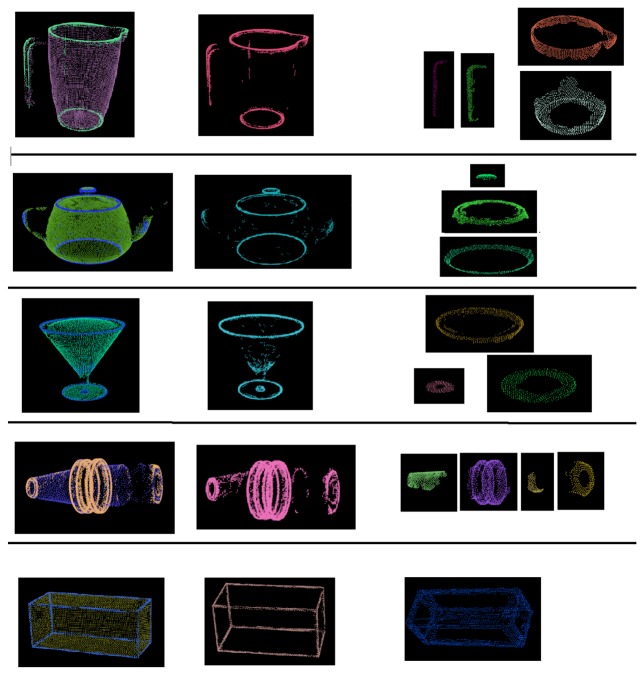
Surface point clouds (**left**) and cumulative edges (**middle**) for a few database objects using the autonomous database creation procedure. The sub-parts based on independent cumulative edges are also shown for each object (**right**).

Each database object is divided into sub-parts which are created autonomously in simulation. In line with the exploration procedure, the sub-parts are defined based on global cumulative edges. Each database object is scanned in simulation using a simulated tactile sensor with the edge following exploration strategy explained above. After a cumulative edge is completely explored (no unexplored end-points remain), the sensor is moved to a new position on the surface of the object using the widest unexplored cone exploration strategy (see Section 4.1). This is repeated till a new edge is detected, when the control passes again to the edge following strategy. Scanning the object for a few hundred iterations (of the widest unexplored cone strategy) results in a close to complete representation of the object's surface. The 3D points of each independent cumulative edge together with other object surface points lying within a threshold distance of these edge points form an object sub-part as shown in Figure 9. The complete 6 dof transformation of each sub-part is also stored w.r.t. a fixed object coordinate system.

### 5.3. Software Architecture and Object Recognition

**Figure 10.**
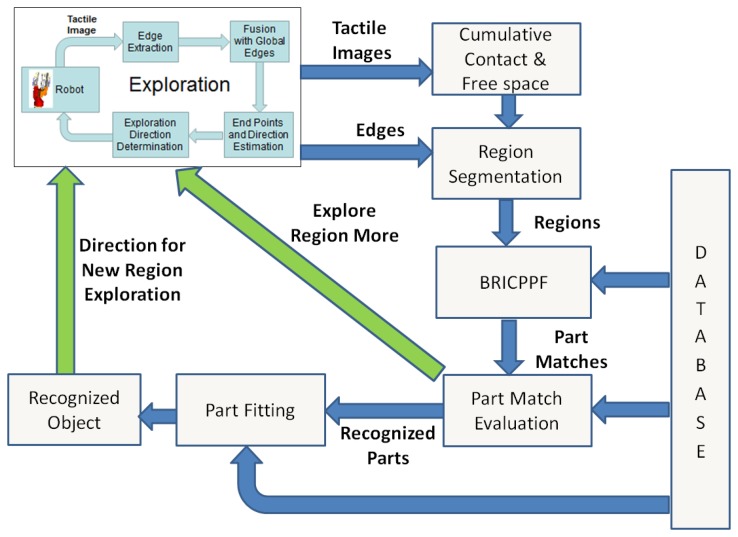
The complete architecture showing the modules and data flow for edge following based exploration and part-fitting based object recognition.

The complete exploration and recognition architecture is shown in Figure 10. The tactile images generated by the *Exploration* node are used for maintaining the cumulative contact and free space point clouds as explained before in Section 4.1. The *Exploration* node also transmits the current cumulative edges in the form of their corresponding contact points. The *Region Segmentation* node segments the contact points in the vicinity of each edge from the cumulative contact point cloud. These points define independent regions for object part matching. These regions are tracked throughout the exploration and recognition procedure. Thus, when multiple edges are connected during exploration, their corresponding regions are also fused together.

The *BRICPPF* node matches each of the regions with the database as explained in Section 4.4. This results in a set of weighed object part matches corresponding to each segmented region. The part matches corresponding to each region are evaluated for satisfactory recognition in the *Part Match Evaluation* node. Two criteria are used for ascertaining whether a region has been satisfactorily matched. First, the amount of region explored should be greater than a threshold fraction (we have chosen 0.3) of the database part that is recognized. This fraction is determined by evaluating $\frac{n_{\text{overlap}}}{N}$ for the object part match with the highest weight (generated by BRICPPF). The database contact point cloud of the recognized part is transformed to the coordinate system of the input region point cloud using the recognized part's 6 dof pose. Then, *n_overlap_* represents the number of points in the database contact point cloud having at least one point of the input region point cloud in its vicinity. *N* is the total number of points in the database contact point cloud. Secondly, the quality of fit for the top ranked part match evaluated using Equation (7) should be greater than a pre-defined quality index. A region is assumed to be satisfactorily recognized only if both of these criteria are satisfied. In this case, the *Exploration* module is asked to terminate the exploration of this part. Otherwise, the *Exploration* module is directed to explore it further as shown by the green arrow in Figure 10.

The part matches corresponding to all the regions are passed to the *Part Fitting* node. The complete part fitting algorithm is presented in Algorithm 2. The algorithm takes a list ℛ of all database part matches corresponding to each region as input. $ℛ=<[p_{0}^{0},p_{1}^{0}\ldots p_{t}^{0}],\ldots ,[p_{0}^{r},p_{1}^{r}\ldots p_{t}^{r}],\ldots ,[p_{0}^{R},p_{1}^{R}\ldots p_{t}^{R}]>$ where $p_{i}^{r}$ is the *i^th^* part match for region *r*. These part matches have been generated by the BRICPPF procedure explained in Section 4.4 and consist of the object index, 6 dof pose and an importance weight. The output state 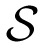 is initialized to be empty in line 2. The procedure in lines 4-9 is repeated *N* times. A region *r* is selected randomly from the set ℛ. In line 5, a match *p^r^* is sampled from the database matches $\left[p_{0}^{r},p_{1}^{r}\ldots p_{t}^{r}\right]$ corresponding to the selected region *r*. The sample is selected using the Cumulative Distribution Function (CDF) created by the importance weights associated with $\left[p_{0}^{r},p_{1}^{r}\ldots p_{t}^{r}\right]$. Thus, a match with higher weight has a greater probability of being sampled than a match with a lower weight. In lines 6 and 7, the object index and 6 dof pose associated with *p^r^* is used to create an object hypothesis. An importance weight is assigned to this hypothesis in line 8 using the *CalculateWeight* function which will be explained below. The newly created hypothesis *s* is added to the state vector 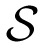 in line 9. Till this point, the object hypotheses in state vector 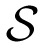 consist of only one region from which they were created. Contributions from new regions are added to the hypotheses in lines 10–14. The addition of each region is tried one by one (line 10). For each region, a maximum of *Q* attempts for addition are made (line 11). In line 12, a hypothesis *s* is selected randomly from 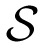. A new hypothesis *s′* is created by adding a new region to the hypothesis *s* using the *AddRegion* function shown in Algorithm 3. *s′* is added to 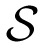 in line 14. Finally, the state 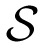 is returned.



**Algorithm 2** Part-Fitting algorithm for object recognition.
1:**procedure** Part Fitting (ℛ)2: 

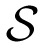
 = Φ3: **for**
*n* = 1 to *N*
**do**4:  Randomly sample region *r* from ℛ5:  Sample match *p^r^* from region matches 
$\left[p_{0}^{r},p_{1}^{r}\ldots p_{t}^{r}\right]$ using CDF of weights6:   (*obj*, *pose*) ← *p^r^*7:  *s* = (*obj*, *pose*, [*p^r^*])8:  *s*(*obj*, *pose*, [*p^r^*], *wt*) ← **CalculateWeight**(*s*)9:  Add *s* to 

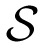
10: **for**
*i* = 1 to *R*
**do**▹ Try to add each region11:  **for**
*j* = 1 to *Q*
**do**12:   Randomly sample *s* from 

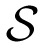
13:   *s′* ← **AddRegion** (*s*, ℛ)14:   Add *s′* to 

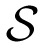
15: Return 

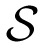



The *AddRegion* function, shown in Algorithm 3, is responsible for adding the matches corresponding to a new region to an object hypothesis *s*. In line 4, for a given hypothesis *s*, a region *r* is randomly sampled from all regions ℛ such that *s* does not already contain matches for *r*. In line 5, a match *p^r^* is sampled from the database matches $\left[p_{0}^{r},p_{1}^{r}\ldots p_{t}^{r}\right]$ for region *r* using the CDF of the importance weights. Lines 6 and 7 check if *p^r^* corresponds to the same object as the hypothesis *s*. Line 8 checks if the 6 dof pose for *p^r^* conforms with the object pose represented by *s* using the *CheckPartPlacement* function. This function checks if the 6 dof pose of the new part falls within a pre-defined threshold of the expected pose for the part assuming the object pose given by *s*. Lines 9–12 add the part *p^r^* to *s* if *CheckPartPlacement* succeeds. The new quality of the hypothesis is calculated using the *CalculateWeight* function and the modified hypothesis is returned.



**Algorithm 3** Algorithm for adding a new region to a hypothesis.
1:**procedure** AddRegion(*s*, ℛ)2: *success* = *false*3: **for**
*i* = 1 to *K*
**do**4:  Randomly sample region *r* from ℛ such that *s* does not contain *r*5:  Sample match *p^r^* from 
$\left[p_{0}^{r},p_{1}^{r}\ldots p_{t}^{r}\right]$ using CDF of weights6:  **if**
*GetObjectIndex*(*p^r^*) != *GetObjectIndex*(*s*) **then**7:   continue8:  *success* = **CheckPartPlacement**(*s*, *p^r^*)9:  **if**
*success* is *True*
**then**10:   Add *p^r^* to *s*11:   *s* ← **CalculateWeight**(*s*)12:   break13: Return *s*


The *CalculateWeight* function calculates the quality *weight_s_* of hypothesis *s* and is given by Equation (11). For each region *r* = [1, *R*] contained in *s*, contributions are taken from the importance weight *W_p_^r^* of the part match *p^r^*; the ratio of the number of points in the input contact point cloud corresponding to region *r* (*pts_r_*) to the maximum number of points in any region input point cloud from 1 to *R*; and the pose fitting error ${\text{Error}}_{p^{r}}^{s}$ associated with *p^r^*.

(11) $${\text{weight}}_{s}=\sum_{r=1}^{R}W_{p^{r}}\frac{{\text{pts}}_{r}}{\underset{1\leq r\leq R}{\max}({\text{pts}}_{r})}{\text{Error}}_{p^{r}}^{s}$$

The pose fitting error ${\text{Error}}_{p^{r}}^{s}$ is given by Equation (12). For each of the six dofs, the difference between the expected position of the part *p^r^* and its actual position is calculated and is given by *δ_dof_. thresh_dof_* represents a pre-defined threshold beyond which the part fitting was rejected in the *CheckPartPlacement* function line 8 of Algorithm 3.

(12) $${\text{Error}}_{p^{r}}^{s}=\prod_{\text{dof}=1}^{6}\frac{\delta_{\text{dof}}}{{\text{thresh}}_{\text{dof}}}$$

The *Part Fitting* node in Figure 10 outputs a set of possible object and pose hypotheses along with their importance weights. The hypothesis with the highest weight is assumed as the recognized object with maximum belief. The object part not currently contained in this hypothesis is determined to be the next region of exploration. A point on this part's database contact point cloud (transformed appropriately w.r.t. the object pose given by the hypothesis) is randomly selected. The position of this point is passed to the *Exploration* node as the next direction for exploration as shown in Figure 10.

## 6. Experiments and Results

Extensive experimentation was conducted in underwater and ground based environments for validating the BRICPPF based object recognition algorithms. Simulation experiments were used for validating the edge following and part fitting based recognition algorithms.

### 6.1. Experiments for BRICPPF Validation

#### 6.1.1. Underwater Tactile Data Collection

**Figure 11.**
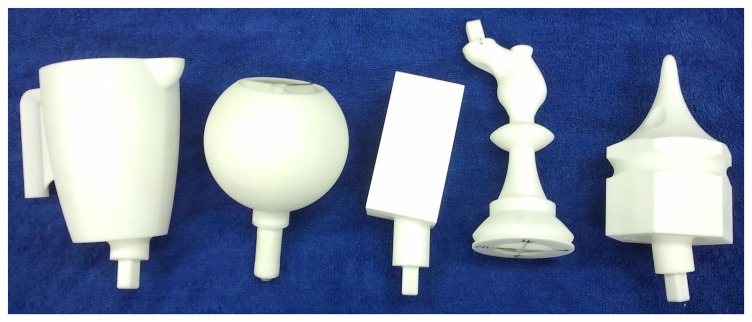
Database objects printed using a rapid prototyping machine for hardware tests.

**Figure 12.**
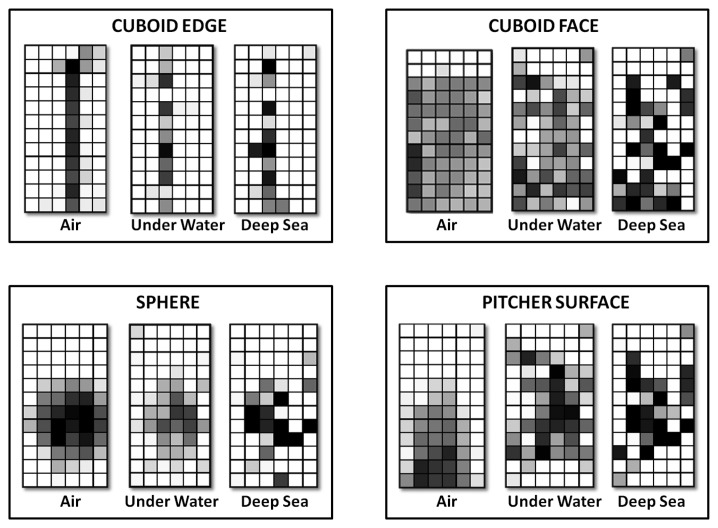
The difference between tactile image quality while exploring standard object surfaces for three different cases: in air; under water; under water at high ambient pressure. Darker shades represent greater foam compression.

For experimental validation of the algorithms, five objects from the object database (Figure 7) were manufactured using a rapid prototyping machine. These objects are shown in Figure 11. The objects have been designed such that they can be fixed onto external fixtures using screws. For underwater experiments, the objects were rigidly mounted onto fixtures inside water. The SeeGrip gripper was attached at the end-effector of the Orion7P manipulator (Figure 2). Data was collected by manually steering the gripper to random positions on the object's surface and closing the grasp. The motion of the manipulator and the gripper takes around 1 min for a manually steered grasp. During the underwater experiments, only one fiber-optic tactile sensor at the distal link of the middle finger (shown in Figure 1) was available for generating contact information. The kinematics of all three fingers were used for generating free space information. At least 50 random grasps were used to explore each of the five objects using this procedure. Some tactile images collected with the middle finger tactile sensor during these experiments are shown in Figure 12.

#### 6.1.2. Tactile Data Collection in Air

The same printed objects were used for tactile data collection in the air. For ground based experiments, the objects were rigidly mounted onto Bosch profiles. The light weight gripper prepared for the air based experiments was attached at the end-effector of a Mitsubishi PA10 manipulator (Figure 3). Data was again collected by manually steering the gripper to random positions on the object's surface and closing the grasp. For ground based experiments, all six fiber-optic tactile sensors were used for generating contact information and the kinematics of the fingers was used for generating free space information. At least 50 random grasps were used to explore each of the five objects using this procedure. Some tactile images collected during these experiments are shown in Figure 12.

#### 6.1.3. Description of Experiments

For evaluating the performance of algorithms on ground and in underwater conditions, object exploration runs were modeled in both environments. An object exploration run is modeled by using tactile measurements from random data collection grasps for each exploration step in the run. Data collection steps of Section 6.1.1. were used for modeling underwater runs and steps of Section 6.1.2. were used for modeling ground based runs. One exploration run consists of 40 exploration steps, and the results are averaged over several complete exploration runs for each object.

*Object Recognition Rate*: At each exploration step, the object recognition rate represents the percentage of times (over multiple complete exploration cycles) the correct object was identified as the top ranked match.

*Pose Estimation Error*: The pose estimation error for each exploration step is also averaged over multiple exploration runs. It is computed as the average euclidean distance between points of the database object contact point cloud at the actual object pose and the points of another similar cloud at the estimated 6D pose. The error is computed only if the correct object is recognized at an exploration step. At every step, the error is bound at a maximum value of 4 cm in case no correct object could be detected for any exploration run. This approach of calculating the 6 dof pose estimation error gives a much better estimate as compared to centroid distance errors. Centroid distance errors can be ambiguous in cases when the centroids of the objects at two different poses are coincident while one of them is rotated around an axis passing through the centroid.

#### 6.1.4. Performance of Batch RANSAC in Underwater Environments

The Batch RANSAC based object recognition and localization algorithm discussed in Section 4.3 was evaluated using the tactile data collected from underwater experiments in Section 6.1.1. for the *Pitcher* object. Search radius of 7 mm was used for surface normal estimation and 10 mm was used for feature calculation. These dimensions have been chosen since the maximum possible radius of a contact patch generated by the sensor is 15 mm. The database consists of 45 objects. For database pruning *W* = 3,000, for contact error estimation *d_outlier_* = 20 cm were used.

**Figure 13.**
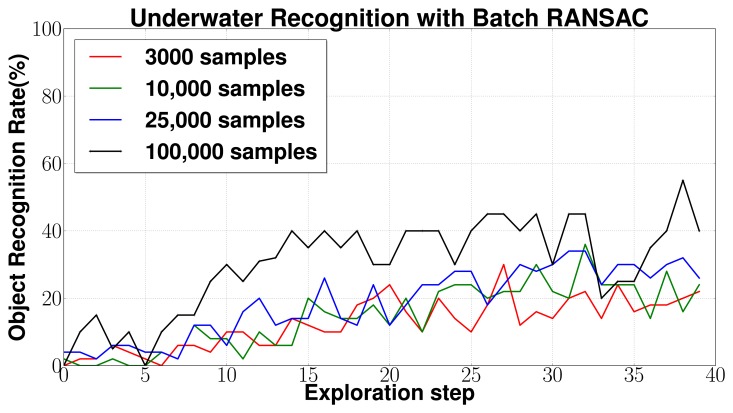
Underwater Object Recognition Performance of the Batch Random Sampling and Consensus (RANSAC) algorithm for object Pitcher with a 45 object database. Results shown with varying number of samples for RANSAC and are averaged over 50 exploration runs.

Performance was evaluated for different combinations of parameters *N*(number of triplet samples from input) and *R*(number of samples in the pruned database for each input triplet). *N* * *R* defines the total number of samples used in RANSAC. Figure 13 shows the results for (a) *N* = 15, *R* = 200 (3,000 samples); (b) *N* = 50, *R* = 200 (10,000 samples); (c) *N* = 50, *R* = 500 (25,000 samples); (d) *N* = 100, *R* = 1, 000 (100,000 samples). The results are averaged over 50 complete exploration cycles each consisting of 40 exploration steps represented by the horizontal axis. The plots show that the object recognition rate increases with increasing amount of object surface being explored. Also, as expected, increasing the number of samples increases the relative recognition rates. However, object recognition rates are unstable and only moderate even after a large part of the object has been explored, even for *case d* with 100,000 samples. The average computation times vary from 2 to 8 *s* for *case a* depending on the number of points in the input point cloud which increases steadily with increasing measurements. Average computation times were 8 to 25 *s* for *case b*, 12 to 40 s for *case c*, and 35 to 100 s for *case d*, all on a Quad-core, Intel I—7 processor with 8 GB RAM.

#### 6.1.5. Performance of BRICPPF in Underwater Environments

For the BRICPPF algorithm, number of particles *n_p_* = 100 and re-sampling parameter Ψ = 80% were used. For free space estimation *s_objmax_* = 20 cm, *res_free_* = 4 cm, *d_thresh_* = 2 cm and λ = 0.02 were used.

**Figure 14.**
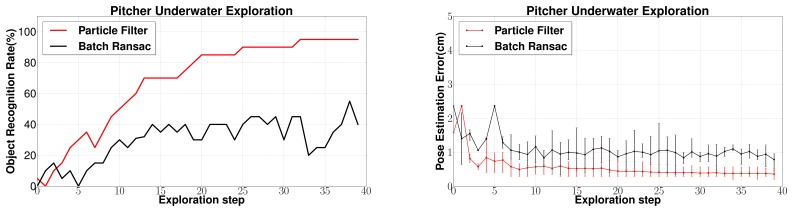
Recognition Rate (left) and Average Pose Estimation Error with 95% confidence intervals (right) comparisons between BRICPPF and Batch RANSAC for object Pitcher. Database consists of 45 objects, and results are averaged over 20 exploration runs.

(a) *Comparison with Batch RANSAC*: Figure 14 shows the performance comparison between BRICPPF and Batch RANSAC. Similar to the previous cases, the tactile data collected from exploring the object *Pitcher* under water was used with 45 database objects. The results are averaged over 20 complete exploration cycles each consisting of 40 exploration steps represented by the horizontal axis. Comparisons are drawn between *case d* (Section 6.1.4.) with 100,000 samples of Batch RANSAC algorithm while for the particle filter algorithm only 3,000 samples are used for its Batch RANSAC component. For the pose estimation error, the average error over 20 runs is plotted along with 95% confidence intervals.

As can be seen in Figure 14, the object recognition rates are low in the beginning of exploration but increase gradually with greater surface of the object being explored. The recognition rates using BRICPPF are much higher (more than double) and show a steady increase with increasing tactile measurements as compared to the unstable behavior of Batch RANSAC. Around 90% success rates could consistently be achieved after only 20 exploration steps. Similar improvements can also be seen in 6 dof pose estimation errors. BRICPPF leads to pose estimation errors below 0.5 cm while for Batch RANSAC, errors are around 1 cm even towards the end of exploration. The average computation times for BRICPPF vary from 19 to 80 s (between 1st and 40th exploration steps). These are comparable to computation times of *case d* (Section 6.1.4.) which shows equivalent computation effort for an adequate performance comparison. As before, results are generated on a Quad-core, Intel I—7 processor with 8 GB RAM.

**Figure 15.**
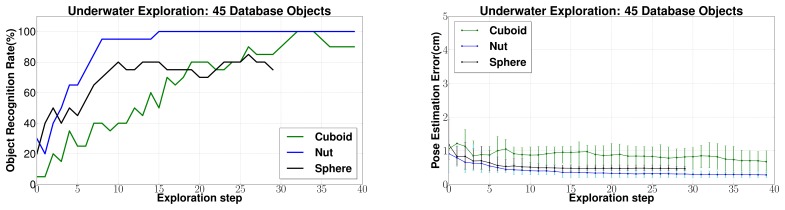
Recognition Rate (**left**) and Average Pose Estimation Error with 95% confidence intervals (**right**) using BRICPPF for objects Cuboid, Nut and Sphere. Database consists of 45 objects, and results are averaged over 20 exploration runs.

**Figure 16.**
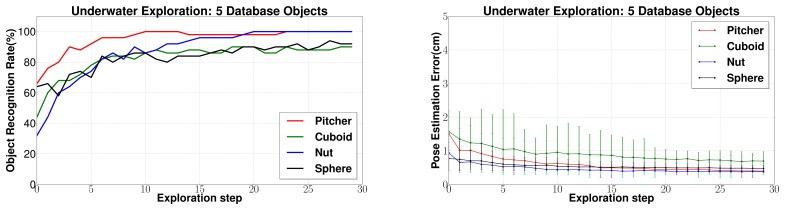
Recognition Rate (**left**) and Average Pose Estimation Error with 95% confidence intervals (**right**) using BRICPPF for four different objects. Database consists of five objects, and results are averaged over 50 exploration runs.

(b) *Results for other objects*: Object recognition and pose estimation results for *Cuboid*, *Sphere* and *Nut* objects are presented in Figure 15. As discussed previously for the *Pitcher* object, these results have also been computed using tactile data collected from the underwater experiments. For all these cases, the recognition rate increases steadily and pose estimation error decreases steadily using sequential state estimation methodology. 80 to 100% object recognition rates were achieved after 15–20 exploration steps. Also, the 6 dof pose estimation error was estimated to be well below 1 cm for all objects.

(c) *Changing database size*: Next, the effect of changing the size of the database is evaluated. In Figures 14 and 15 a database of 45 objects has been used to conform to a practical situation where a large database is required. However, for comparison with other approaches in literature, it makes sense to present results for a smaller database of five objects. The results are shown in Figure 16. Reduction in the database size leads to a large improvement in performance as compared to the case with a large database. This is justified by the reduction in the search space. Eighty to 100% object recognition rates were achieved only after five exploration steps for all the objects. Also, the average 6 dof pose estimation error was estimated to be well below 1 cm for all objects.

#### 6.1.6. Experiments in Ground Based Environments

**Figure 17.**
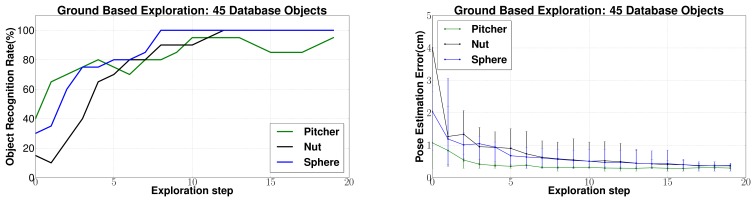
Ground based Recognition Rate (**left**) and Average Pose Estimation Error with 95% confidence intervals (**right**) using BRICPPF for objects Pitcher, Nut and Sphere. Database consists of 45 objects, and results are averaged over 20 exploration runs.

Similar experiments were conducted with tactile data collected from the ground based experiments. The recognition and localization results for three different objects are shown in Figure 17 using a database of 45 objects. As seen for the underwater experiments, the recognition rate increases steadily and pose estimation error decreases steadily with increasing object surface being explored. 80% to 100% object recognition rates were achieved after only 5–7 exploration steps. Also, the 6 dof pose estimation error was estimated to be well below 1 cm for all objects. This is because of the increased quality of contact tactile data in the case of ground based exploration as compared to underwater exploration which can also be seen in Figure 12. Also, the EEF positioning error associated with the Mitsubishi PA10 manipulator used for ground tests is much lesser as compared to that of Orion7P manipulator for underwater experiments.

**Figure 18.**
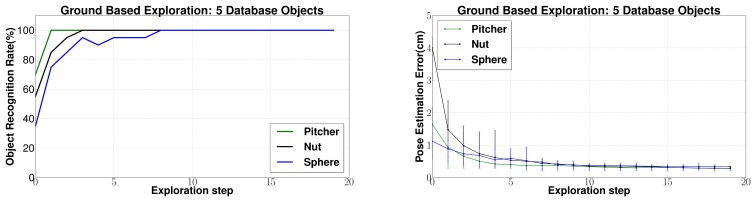
Ground based Recognition Rate (**left**) and Average Pose Estimation Error with 95% confidence intervals (**right**) using BRICPPF for objects Pitcher, Nut and Sphere. Database consists of five objects, and results are averaged over 20 exploration runs.

The results with a smaller database of five objects is shown in Figure 18. An even better performance can be seen in this case and 80% to 100% object recognition rates were achieved only after three exploration steps for all the objects. Also, the average 6 dof pose estimation error was estimated to be below 0.5 cm for all objects.

### 6.2. Experiments for Part Fitting Validation

#### 6.2.1. Description of Experiments

The experiments for validating the edge following based exploration strategy (Section 5.1) and part fitting based recognition algorithms (Section 5.3) were conducted in simulation. A tactile sensor simulation was created in the OpenRAVE simulation environment [51] and the software architecture was created using the Robot Operating System (ROS) framework [52]. The tactile sensor array consists of 324 sensel elements distributed in a 27-by-12 arrangement as shown in Figure 8 in line with a real high resolution sensor module currently under development. The database consists of 20 objects with well defined edges. Five objects from the database are shown in Figure 9.

#### 6.2.2. Object Part Recognition

**Figure 19.**
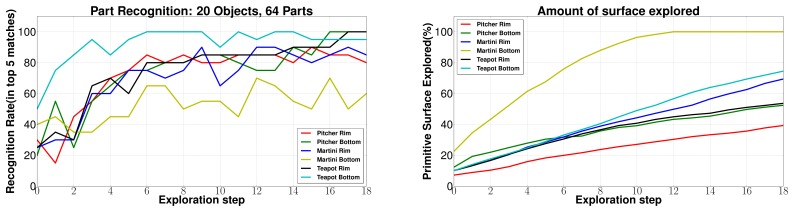
Part recognition rate (**left**) and Average amount of surface explored (**right**) using edge following algorithms and BRICPPF. Results are averaged over 20 exploration runs.

The complete edge following and recognition architecture (Section 5.3) was validated via extensive experimentation in simulation. Several complete exploration and recognition runs were conducted on different objects which validated the robustness of the architecture. The database of 20 objects consists of a total of 64 object parts. The performance of BRICPPF for part matching was analyzed. Tactile data was collected using the edge following methodology on many different object parts. The results for recognition rate *versus* the number of exploration steps are shown in Figure 19. The recognition rate at a particular step represents the percentage of times (over 20 complete runs) at least one correct part was ranked in the top five matches. The average amount of the part surface explored (averaged over 20 runs) is also plotted on the right side of Figure 19 with respect to the number of exploration steps. It can be seen that around 80% success in part recognition could be achieved in 8 exploration steps which corresponds to 40% of the part surface being explored.

#### 6.2.3. Part Fitting based Object Recognition

**Figure 20.**
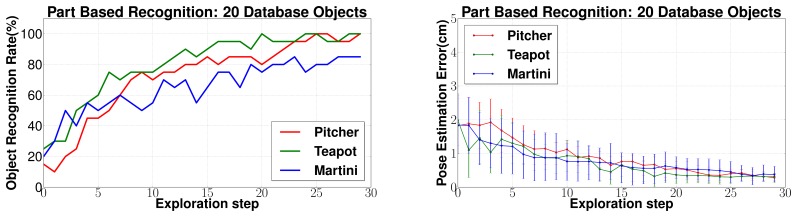
Object Recognition Rate (**left**) and Average Pose Estimation Error with 95% confidence intervals (**right**) using edge following and part based object recognition for three different objects. Database consists of 20 objects, and results are averaged over 20 exploration runs.

For part fitting based recognition (Section 5.3), the parameter values of *N* = 100, *t* = 50, *R* = 100, *Q* = 50 were used for Algorithm 2 and *K* = 100 for Algorithm 3. The object recognition and pose estimation error results for three objects Pitcher, Teapot and Martini glass (as shown in Figure 9) are shown in Figure 20. It can be seen that 80%–100% recognition success rates were achieved in 15–20 exploration steps, and less than 1 cm pose estimation errors were also achieved.

## 7. Discussion

### 7.1. Discussion

**(a) Performance of BRICPPF:** The experiments prove that the BRICPPF methodology results in large object recognition and localization performance improvements over the standalone Batch RANSAC based database matching methodology. The results in Figure 13 show that Batch RANSAC alone does not yield high recognition performance even after several measurements. Further, even increasing the computational effort by increasing the number of samples to 100,000 samples does not result in major improvements in the performance. This is because the success of this approach depends on the selection of the correct object matches, first during the feature based database pruning step, and then on finding the correct triplet matches during registration. Thus, even if the database is pruned correctly, unless all possible object and 6 dof pose hypotheses are tested comprehensively, there is a possibility of the correct object and pose not being detected. Further, since the Batch RANSAC algorithm does not involve sequential tracking of hypotheses, detecting the correct hypothesis at one instance during the course of exploration does not have any influence on the success at a subsequent exploration step. This leads to moderate and unstable object recognition rates even after a large part of the object has been explored.

The ICP augmented sequential state estimation on the other hand ensures that if, at any instance during the course of exploration, a hypothesis is detected in the vicinity of the correct object and the correct pose, it will be tracked and successively evolved to the correct pose. This results in high recognition rates (Figure 14). These comparisons have been drawn with a high number of samples (100,000) used in Batch RANSAC to ensure comparable computational times. The ICP and Batch RANSAC based resampling efficiently allows managing the high dimensionality of this 7 dimensional problem, which is otherwise computationally prohibitive for standard Bayesian methods. Therefore, only 100 particles are required in the complete state space. The use of feature based RANSAC within the sequential state estimation also allows the handling of large databases. While the 45 object database consists of more than 90,000 object surface feature points, feature based pruning allows the database to be pruned to only 3,000 points for each input sample which represents only 3.33% of the database.

With a large database of 45 objects, 80%–100% recognition rates were achieved within 15–20 grasps for all four objects for the underwater experiments. Pose estimation errors of around 5 mm were also achieved. This is quite acceptable for under water applications, given the inaccuracies in industrial under water manipulator positioning. For the ground based experiments, 80% to 100% object recognition rates were achieved after only 5–7 exploration steps. This is because of the increased quality of tactile data in the case of ground based exploration as compared to underwater exploration which can also be seen in Figure 12. Secondly, all six tactile sensors were used for the ground based experiments as compared to underwater experiments where only one sensor was used for contact data collection. Free space, however was estimated using all three fingers in both cases. However, while all six sensors were used for data collection, not all were in contact with the object at every data collection step. The third reason for performance improvements in ground based experiments is that the EEF positioning error associated with the Mitsubishi PA10 manipulator used for ground tests is much lesser as compared to that of Orion7P manipulator used for underwater experiments.

**(b) Performance of Edge Following and Part Fitting based Recognition:** The results for object part recognition using BRICPPF, shown in Figure 19, are in line with the other BRICPPF results discussed above. Object part recognition was achieved with 80% success in about 8 exploration steps which corresponds to 40% of the part surface being explored. This is with a database consisting of 64 object parts, with several parts like object rims and bottoms having very similar shapes. Also the amount of data collected using the edge following exploration approach is limited to only the edge regions. Thus, a large portion of the tactile sensor surface remains untouched in a data collection step. The attainment of high success rates even in this case proves that edges are in fact more suited for distinguishing between objects as compared to the general object surface.

The close integration of the object exploration and recognition was proven to be successful and using only a single tactile sensor, 80%–100% object recognition success rates were achieved in 15–20 exploration steps. It should be noted that on an average, around 12 exploration steps are devoted towards satisfactorily recognizing a single object part. As soon as a second object part is explored, the recognition rate quickly increases after 15 exploration steps.

This approach is based on the biological principles of exploration and recognition. The performance of this approach is quite satisfactory but no clear improvements could be seen as compared to the BRICPPF approach. This approach is also limited to objects with well defined edges, and can be used optimally in situations where only a single tactile sensor is available.

**(c) Comparisons with other approaches:** In this article, haptic underwater object recognition and localization has been addressed for the first time in literature. Comparisons can be drawn with other approaches for terrestrial environments. The most relevant previous work [18] comprises of the application of particle filters and histogram filters for 3 dof localization of 2D objects using a dense array of tactile sensors similar to the SeeGrip sensors. Occupancy maps of objects built with real sensor based ground truth are used as a database. The histogram filter with 10,000 bins was able to successfully achieve 100% recognition rates with nine sensor readings with a database of five objects. The objects were localized to within 1.3 mm position error. With our BRICPPF approach, we were able to achieve 80%–100% recognition success with 5–10 grasps for a database of five objects in the underwater case and in only three exploration steps for the ground based environments. Pose estimation error was around 5 mm. It should be noted again that we deal with 3D objects, and consider complete 6 dof localization. Also, for underwater scenarios, the position errors associated with manipulator systems and tactile sensors are much greater than ground based systems.

Another approach [16] achieves 80%–100% recognition with a seven object database using 5–6 palpations. This approach, however, performs recognition directly in the haptic space using gripper kinematics and tactile sensor data without building a representation of the object. Thus, localization is not addressed. Similarly, another approach [14] uses bag of features for object recognition and results in 85% recognition success with a database of 21 objects in 10 grasps. Again, it does not deal with object localization. Also, both these approaches depend on the ground truth data collected from real sensors for creating a training data set. Another approach [19] presents 6 dof localization of a known 3D object using a tactile probe. It uses an annealed Particle filter and gradually scales precision to manage large dimensional spaces and results in localization accuracy of 5 mm position and 3 degree rotation in around 12 probes. Object recognition adds another dimension to the 6 dof localization problem, and the performance of this approach is expected to degrade considerably for this case.

The widest unexplored cone based exploration strategy presented in this article diverts attention to most unexplored regions and is based on the assumption that gathering new information is always advantageous. Our approach does not use polygonal structures of objects (as compared to [20]) and can handle objects of any complicated shapes. Also, the actions are not selected from a pre-defined set of limited actions, and motion plans are generated autonomously for approaching the maximum unexplored object surface area. For more efficient exploration, the edge based exploration strategy was tried which is also efficiently tied to the current object recognition state. The task driven exploration strategy presented in [20] and information gain strategy [14] are other possible ways of providing an efficient link between exploration and recognition. However, these approaches will have to be adapted to tackle object recognition and 6 dof pose estimation.

**(d) General Discussion:** The problem of static object recognition for structured underwater and ground based scenarios has been tackled efficiently using BRICPPF, which is a sequential state estimation methodology with an ICP augmented particle filter used in combination with Batch RANSAC based database matching. It has been shown to work with a large database of objects, objects of complex shapes, and can work with any configuration of the gripper with any number of tactile sensors. It has also been proven that it is not necessary to create the database from the actual ground truth which is necessary for a reasonably autonomous scenario. The approach also utilizes both contact and free space information. It can also incorporate other sensor modalities within its probabilistic framework (see Equation 7). The computational time of less than 1 min (up to the 20th exploration step) for each exploration step of the BRICPPF is also reasonable. This is quite comparable to the time taken by the manipulator to move to a new exploration position and can therefore be done in parallel to the robot motion.

The widest unexplored cone exploration strategy is also very effective in dealing with objects of complicated shapes and can work for any configurations of the manipulator and gripper systems. It can also be argued that a complicated exploration procedure closely tied with recognition state might not even be necessary for optimal haptic recognition, especially when only a few exploration steps are enough for object recognition. We have proven that allowing the exploration to function independent of the current state of recognition works fine with robust object recognition being achieved in only a few exploration steps. In less than 10 exploration steps, exploring new regions on the object surface might be sufficient and even the most optimal exploration technique since greater information about the shape and size of the object can be gathered using this approach. We are heading towards a future where robot appendages will be equipped with a tactile skin consisting of a large number of tactile sensors. The BRICPPF approach can be directly applied to such a situation for robust object recognition, object localization, robot self localization in the environment, and for robot calibration.

The results presented in the current article can be considered as a step towards achieving autonomy in under water and ground environments. A similar approach has been presented earlier for movable object localization with simulated data [2] which will also be tried with real hardware data in the future. Also, the performance of the edge following and part fitting based object recognition has been shown to be quite satisfactory. It could be compared to the performance of actual human haptic exploration and recognition in the future. In such an experiment, it is necessary to force the human subjects to use limited dexterity similar to a robot system. Further, rapid advancements are being made in space exploration [53,54]. The presence of extraterrestrial dust is a huge bottleneck for robust autonomous space exploration [55]. The haptic exploration and recognition methods discussed in this article are directly applicable to space exploration as well.

## 8. Conclusions

Two approaches for recognition and 6 dof localization of pre-known objects using tactile data for structured ground based and underwater applications was presented. In the first approach, a tactile sensor system along with an unexplored region seeking exploration strategy enabled the representation of tactile data as locally dense point clouds. Thus, state of the art point cloud matching techniques from range sensing literature were selected, adapted and extended for object recognition. For adding robustness, a sequential state estimation methodology based on ICP augmented particle filters, called BRICPPF was presented. The feature based RANSAC and ICP allowed efficient management of high dimensional spaces within the particle filter framework. Free space information managed using a 3D occupancy map led to an accelerated recognition process. The methodology is independent of the acquisition of ground truth data for database creation and the database can be created using a simulated laser scanner. New objects can also be autonomously added to the database. Tactile data collected from ground based and underwater experiments was used to validate the algorithms for static object recognition and complete 6-dof localization. For underwater experiments, recognition performance of between 80%–100% was achieved for four different objects within 20 exploration steps for a 45 object database. 6 dof pose was estimated to an accuracy of 5 mm. The experiments showed over 100% improvements by using the sequential state estimation techniques over RANSAC and feature based database matching. For a smaller database of five objects, similar performance could be achieved within 5 grasps. For ground based experiments, similar success rates were achieved within 5–7 exploration steps for a database of 45 objects and with only three exploration steps with a smaller database of five objects.

The second approach is biologically inspired and is based on a close integration of exploration and recognition strategies. An edge following strategy is used for exploring an object's sub-part continuously and only until it is satisfactorily recognized. BRICPPF is used for object part's recognition from the database. The exploration module is either directed to explore the currently explored object part further, or it is directed to explore a new part depending on the current state of recognition. A recognition by parts approach is developed which spatially fits the identified object parts together to recognize the complete object and determine its 6 dof pose. This approach is shown to work well with objects with well-defined edges and is particularly suited for applications with only a single tactile sensing unit. It is validated via simulation experiments and results in 80%–100% recognition success in about 15 exploration steps.

To our knowledge, haptic recognition and 6 dof pose estimation for structured underwater environments has been presented for the first time in literature. The results presented can be considered as a step towards achieving autonomy in under water and ground based environments. A similar approach has been presented earlier for movable object localization with simulated data [2] which will also be tried with real hardware data in the future. Also, the performance of the edge following and part fitting based object recognition has been shown to be quite satisfactory. It could be compared to the performance of real human haptic exploration and recognition in the future, where the subjects are forced to use limited dexterity similar to a robot. In the under water experiments presented, only one tactile sensor was used for object exploration. The recognition performance is expected to improve with the use of the remaining five tactile sensors on the SeeGrip gripper. It is expected that robust recognition can be achieved within 4–5 grasps of such a system.

The interface between the object exploration and recognition modules can also be improved in the future. Presently, the state of recognition directs exploration towards unexplored object sub-parts based on the object hypothesis with the highest belief. This can be improved by considering multiple high probability object hypotheses and diverting exploration towards regions that would minimize conflicts between these hypotheses. Another possible direction could be to use exploration to directly confirm the recognized object and its pose hypotheses. This could be achieved by directly comparing the predicted and measured tactile data and using it for updating the state beliefs.

The results presented in this article only demonstrate the absolute object recognition capabilities of the algorithms. However, their performance is closely related to the size of the object database and the amount of disparity between various database objects. Thus, a more detailed analysis like understanding the reasons for wrong matches or evaluating confusion matrices amongst the correct and false object matches could be conducted in the future.

## Figures and Tables

**Figure A1.**
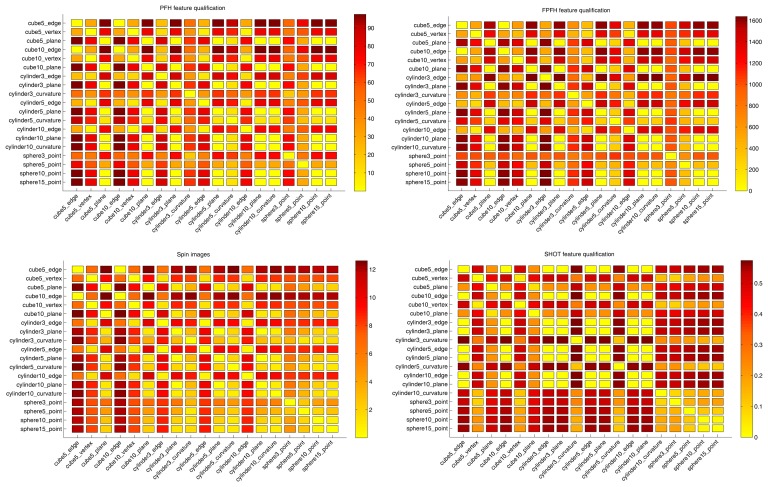
Discriminative characteristics of different features.

## Appendix

### A. Feature Selection

A variety of features and signatures have been presented in literature that quantify the local or global shape of objects. For tactile sensing applications it is necessary to choose features that are pose invariant and have a high discriminative power that enables reliable point correspondence during database matching. Further, the feature computation should be quick to enable online computation of input cloud features. The descriptiveness of Spin Images [56], Signature of Histograms of Orientations (SHOT) [57], Persistent Feature Histograms (PFH) [58] and Fast Point Feature Histograms (FPFH) [45] was compared for selecting the most appropriate feature. A database of 9 different objects (two cubes of sides 5 cm and 10 cm; three cylinders of radii 3 cm, 5 cm and 10 cm and height 5 cm; and four spheres of radii 3 cm, 5 cm, 10 cm and 15 cm) was prepared for comparison. These objects consist of 19 basic shapes representing the edges, vertices and planar faces of the cubes; edges, curved surfaces and planar surfaces of the cylinders; and surfaces of the spheres.

All four features of all relevant regions on the objects' surfaces were evaluated. The implementations of all four features in the Point Cloud Library [59] were chosen for comparison. The default parameters as suggested by the authors were used. For Spin Images, the suggested parameters consist of 8 bins along one dimension, 0 degree support angle, and 153 total dimensions for feature histogram. For SHOT, the suggested parameters consist of 10 shape bins, 32 grid sectors, 28 angular sectors, 0 lrf radius and 352 dimensions of the feature vector. For PFH, suggested parameters are 125 feature dimensions, and for FPFH, 33 feature dimensions were used. For normal estimation, search radius of 1 cm was used and for feature estimation, neighborhood search radius of 1.5 cm was used which corresponds to the maximum expected contact patch radius of the fiber optic sensor (3 cm width).

For comparison of descriptiveness, for each feature, the euclidean distance between the feature vector values for all pair combinations of the 19 shapes was evaluated. These distances were plotted using heat maps as shown in Figure A1. The map for each feature varies from bright yellow to dull red color, with the former representing a negligible difference and the latter representing a large estimate of distinctiveness. Thus, the capabilities of all four features in distinguishing between two given shapes can be judged by the color of the corresponding bin.

The SHOT features seemed to detect only negligible difference between curved cylindrical edges and straight cube edges (the corresponding bins are yellow in color). Also, the difference between large spheres and planar surfaces is large, which seems incorrect, as for the given parameters a sphere of 15 cm radius has a curvature comparable to a planar surface. The performance of FPFH and Spin Images seemed quite comparable except for a few cases. FPFH was able to distinguish between curved cylindrical surfaces of different radii much better than Spin Images. Similarly, FPFH can detect the difference between cylindrical edges of different curvatures better than Spin Images. FPFH is also able to distinguish between spheres of different radii better than Spin Images. The performance of FPFH and PFH are very similar with no critical differences. However, FPFH has a much lesser computational time and uses only 33 dimensions as compared to PFH with 125 dimensions and Spin Images with 153 dimensions. Thus, FPFH was chosen as the most appropriate feature for the tactile sensing application.
